# Mitochondrial lineage sorting in action – historical biogeography of the *Hyles euphorbiae* complex (Sphingidae, Lepidoptera) in Italy

**DOI:** 10.1186/1471-2148-13-83

**Published:** 2013-04-18

**Authors:** Michael B Mende, Anna K Hundsdoerfer

**Affiliations:** 1 Museum für Tierkunde, Senckenberg Naturhistorische Sammlungen Dresden, Königsbrücker Landstr. 159, Dresden, 01109, Germany; 2 Biodiversität und Klima Forschungszentrum (BiK-F), Senckenberganlage 25, Frankfurt am Main, 60325, Germany

**Keywords:** Historic DNA, Ancient DNA *sensu lato*, Natural history collections, mtDNA, Phylogeography, Incomplete lineage sorting, Genetic drift, Climate change

## Abstract

**Background:**

Mitochondrial genes are among the most commonly used markers in studies of species’ phylogeography and to draw conclusions about taxonomy. The *Hyles euphorbiae* complex (HEC) comprises six distinct mitochondrial lineages in the Mediterranean region, of which one exhibits a cryptic disjunct distribution. The predominant mitochondrial lineage in most of Europe, *euphorbiae*, is also present on Malta; however, it is nowadays strangely absent from Southern Italy and Sicily, where it is replaced by '*italica*'. A separate biological entity in Italy is further corroborated by larval colour patterns with a congruent, confined suture zone along the Northern Apennines. By means of historic DNA extracted from museum specimens, we aimed to investigate the evolution of the mitochondrial demographic structure of the HEC in Italy and Malta throughout the Twentieth Century.

**Results:**

At the beginning of the Twentieth Century, the European mainland lineages were also present at a moderate frequency in Southern Italy and Sicily. The proportion of '*italica*' then steadily increased in this area from below 60 percent to near fixation in about 120 years. Thus, geographical sorting of mitochondrial lineages in the HEC was not as complete then as the current demography suggests. The pattern of an integral '*italica*' core region and a disjunct *euphorbiae* distribution evolved very recently. To explain these strong demographic changes, we propose genetic drift due to anthropogenic habitat loss and fragmentation in combination with an impact from recent climate warming that favoured the spreading of the potentially better adapted '*italica*' populations.

**Conclusions:**

The pattern of geographically separated mitochondrial lineages is commonly interpreted as representing long term separated entities. However, our results indicate that such a pattern can emerge surprisingly quickly, even in a widespread and rather common taxon. We thus caution against drawing hasty taxonomic conclusions from biogeographical patterns of mitochondrial markers derived from modern sampling alone.

## Background

Mitochondrial genes are among the most commonly used markers for phylogeographic and taxonomic studies [1-6]. However, there are many pitfalls that can mislead the interpretation of phylogenies and/or phylogeographies based on mitochondrial genes, such as nuclear pseudogenes (‘numts’) [4,7], introgression through hybridization or incomplete lineage sorting [2,4]. The latter is commonly encountered and a particular problem for disentangling relatively young species [4], where different mitochondrial lineages do not necessarily coincide with current evolutionary entities. Nevertheless, the pattern of currently geographically separated lineages is commonly interpreted as being stable in the long term [6,8] and thus useful to delimit taxa or deduce conservation units [1,8-11].

The development of methods to extract DNA from ancient and historical samples [11-14] has opened up the possibility to directly study the evolution of demographic and/or biogeographical patterns, i.e. changes in these patterns over time. Thomas *et al.*[15] found no significant change in the distribution patterns of mitochondrial lineages in stable large populations of kangaroo rats during the Twentieth Century. In contrast, the studies of Pergams *et al.*[16] and Goldstein & DeSalle [17] did reveal that such patterns can be altered significantly in a comparable time span. For instance, recent habitat fragmentation led to the extinction of populations with intermediate haplotypes and thus simulated a pattern of long-term separated lineages in the tiger beetle *Cicindela dorsalis*[17]. Similarly, Leonard *et al.*[18] could show by means of ancient DNA extracted from subfossil bones that a supposed long-term pattern of geographically separated mitochondrial lineages of the brown bear in Alaska had evolved more recently than was previously thought. Consequently, Pääbo *et al.*[11] encouraged further studies on the history of phylogeographic patterns to determine whether modern patterns are recent effects of genetic drift or reflect long-term separation of populations.

In the field of evolutionary genetic studies on museum specimens, only few studies were conducted so far on insect species, e.g. [17,19-22] (also see [12]), although they are a very suitable model group. Especially those insects that are popular among amateur collectors have accumulated a large representation in museums and collections of specimens dating back to the late Nineteenth Century [22], which often equates to more than a hundred generations. Hawkmoths are conspicuous insects characterized by large size and often striking colour. The widespread spurge hawkmoth (*Hyles euphorbiae* complex: HEC) is particularly appealing to collectors due to its great variability in colour pattern [23-25]. Taxonomic delimitations are based on these patterns as genitalia are almost uniform throughout this globally distributed genus, which thus allows (or at least does not prevent) hybridization between even distantly related species [23,26,27]. Accordingly, the species-level taxonomy has been controversial for a long time [23,24,26-29] and, for example, the taxon *Hyles euphorbiae* has accumulated 102 synonyms [30]. A recent molecular study (based on sequences of the mitochondrial genes encoding COI, tRNA leucine and COII) of more than 350 specimens from across the whole range of the HEC revealed that it comprises six distinct mitochondrial lineages in the Mediterranean region [31]. Unexpectedly, three of these lineages were found to conflict with the current taxonomy and thus were given provisional names (cited in quotation marks) [31]. The distribution of the lineages appeared mainly to reflect isolation in refuges during the Ice Ages and subsequent postglacial range expansions [25-27,31]. The southernmost lineage, *tithymali* corresponds closely to the current valid species, *Hyles tithymali*, which occurs in the Macaronesian islands, Northern Africa and a now isolated population in Yemen. Most of continental Europe, from the Caucasus and the Balkan Peninsula to Portugal, the range of the current species *H. euphorbiae*, is occupied sympatrically by the lineages *euphorbiae* and '*enigmatica*'. They were proposed to be colonizers from two Eastern European and/or Central Asian glacial refuges that had expanded simultaneously into large areas of Europe after the end of the last Ice Age [27,31]. The mitochondrial lineage of the endemic *H. cretica* is confined to Crete and the Dodecanese islands. As has been found in many other groups of organisms [5,8,32,33], the Italian glacial refuge gave rise to its own endemic HEC entity, the '*italica*' lineage. It dominates Southern Italy and Sicily but is replaced by *euphorbiae* and '*enigmatica*' northeast of the Northern Apennines. Even though the Southern Italian populations are currently synonymised under *H. euphorbiae*, morphology also suggests a distinct entity. The change of mitochondrial haplotypes in northern Central Italy is congruent with a sharp change in larval colour patterns [25]. Furthermore, subspecies of *H. euphorbiae* have been regularly described from Southern Italy based on the frequent occurrence of a reddish adult forewing pattern variety [34-37] which is absent from Central Europe.

The Maltese endemic, *H. sammuti*, has long been considered a hybrid population between African and European lineages and therefore probably invalid as a species, though nevertheless interesting from an evolutionary perspective [23,26,27,29,31,38]. Paradoxically, it is the European mainland lineage *euphorbiae* that is the major component (over 50%) of the Maltese population, in addition to the ancient endemic '*melitensis*' lineage and migrants of the North African *tithymali*[31]. No ‘*italica’* haplotypes have yet been recorded from this island, despite its proximity to Sicily. This colony of *euphorbiae* haplotypes on Malta is isolated from the *euphorbiae* core population in Central Europe and Northern Italy by about 900 kilometres of intervening land inhabited by '*italica*'.

In the present study, we investigate the evolution of the mitochondrial marker demography of the HEC in Italy and Malta directly by means of historic DNA from museum specimens. Thereby we aim to determine if and/or how the disjunct distribution pattern of the *euphorbiae* lineage and an integral '*italica*' core region evolved during the last century and to propose hypotheses for the underlying mechanisms.

## Results

### Sequence yield

In total, we accumulated samples of 216 relevant specimens from the target area (Additional file 1: Table S1). We successfully amplified all three targeted fragments (237–280 bp in length) of the CO I/II genes from 143 specimens (Additional file 1: Table S1). This corresponds to an amplification success of 66.2% for all target fragments. From a further 20 specimens we were able to sequence only the shortest fragment L, accompanied by stochastic amplification of only one of the longer fragments in eleven of these specimens (Additional file 1: Table S1). Hence, the success rate was considerably higher for fragment L alone (75.5%). However, the success rate differed strongly between collections (see Table 1). PCR success was significantly higher for specimens from museums of higher geographical latitude (*rho* = 0.503; *p* < 0.001). It was lowest for the samples from the Maltese collections, and the MRST, Terrasini, even though the samples from these collections were comparably young (Table 1, Additional file 1: Table S1). Advanced decomposition of specimens of the MRST was further indicated by the occasional amplification of non-target yeast sequences for fragment L. Accordingly, we did not find a significant correlation over all samples between PCR success and lower sample age (*rho* = −0.027; *p* = 0.691). However, if the samples from the Maltese and MRST collections are excluded from the data set, the trend becomes significant (*rho* = −0.301; *p* < 0.001), i.e. PCR success is significantly lower with higher age of samples.

**Table 1:** List of museums and/or private collections that provided samples

**Abbrev.**	**Museum / private collection**	**Contact**	***N***	**Success**	**Age**
**ZMH**	Zoologisches Museum Hamburg	Bernhard Misof, Hans-Georg Riefenstahl, Ralph Peters, Kai Schütte	8	87.5 (100)	1938-1950
**MNB**	Museum für Naturkunde, Berlin	Wolfram Mey, Konrad Ebert	4	100	1884-1885
**NHM**	Natural History Museum, London	Geoff Martin, Ian J. Kitching	36	80.6 (94.4)	1894-1980
**SMTD**	Senckenberg Naturhistorische Sammlungen Dresden, Museum für Tierkunde	Matthias Nuß	2	100	1930-1940
**ZFMK**	Forschungsmuseum Alexander König, Bonn	Dieter Stüning, Ute Heidenreich, Ulrike Kleikamp *	10	60.0 (80.0)	1909-1928
**SMF**	Senckenberg Naturmuseum, Frankfurt/Main	Wolfgang Nässig	6	100	1888-1937
**SMNK**	Staatliches Museum für Naturkunde, Karlsruhe	Robert Trusch	-		
**SMNS**	Staatliches Museum für Naturkunde, Stuttgart	Andreas Zwick, Daniel Bartsch *	10	100	1964-1976
**ZSM**	Zoologische Staatssammlung München	Axel Hausmann	22	72.7 (81.8)	1885-1963
**MWM**	Museum Witt, München	Wolfgang Speidel, Axel Hausmann	10	90.0	1930-1971
**HNHM**	Hungarian Natural History Museum, Budapest	Zsolt Balint	3	66.7	1884-1891
**MTSN**	Museo Tridentino di Scienze Naturali, Trento	Mauro Gobbi	-		
**MCSNM**	Museo Civico di Storia Naturale, Milano	Fabrizio Rigato, Maurizio Pavesi, Michele Zilioli *	17	82.4	1906-1969
**MCVR**	Museo Civico di Storia Naturale, Verona	Roberta Salmaso	6	83.3	1941-1968
**MRSN**	Museo Regionale di Scienze Naturali, Torino	Luca Christiano, Gianfranco Curletti	-		
**MCCI**	Museo Civico di Storia Naturale di Carmagnola	Gianfranco Curletti	-		
**MSNG**	Museo di Storia Naturale “Giacomo Doria”, Genova	Fabio Penati *	17	94.1	1901-1989
**MZUF**	Museo Zoologico „La Specola“, Universita di Firenze	Luca Bartolozzi, Fabio Cianferoni, Francesca Zinetti	11	27.3 (63.6)	1901-1970
**MSNTUP**	Museo di Storia Naturale del Territorio, Universita di Pisa, Calci	Marco Dellacasa, Leonardo Dapporto	2	50.0	1972
**MSNM**	Museo di Storia Naturale del Mediterraneo, Livorno	Emanuela Silvi	-		
**MSNMG**	Museo di Storia Naturale della Maremma di Grosseto		-		
**MCZ**	Museo Civico di Zoologia, Roma	Alberto Zilli	17	64.7 (70.6)	1900-1984
**MRST**	Museo Regionale di Storia Naturale e Mostra Permanente del Carretto Siciliano, Terrasini	Piera Iacobelli, Federico Marrone	14	7.1 (35.7)	1911-1960
**MZUP**	Museo di Zoologia „P. Doderlein” dell’ Universita di Palermo	Maurizio Sara, Federico Marrone	-		
**NMNH**	National Museum of Natural History, Mdina, Malta	John Borg, Aldo Catania *	16	6.3 (12.5)	1953-1981
**pcPS**	Private collection of Paul Sammut, Rabat, Malta	Paul Sammut, Aldo Catania *	3	0	1965-1983
**pcAC**	Private collection of Aldo Catania, Zebbug, Malta	Aldo Catania *	2	0	1983-1986

Collections sorted by latitude of locality; * = specimens sampled by staff of the museum; *N* = number of specimens sampled; *Success* = percentage of PCR success for all three targeted fragments (for the shortest fragment L in brackets); *Age* = age range of the vouchers.

**Figure 1:**
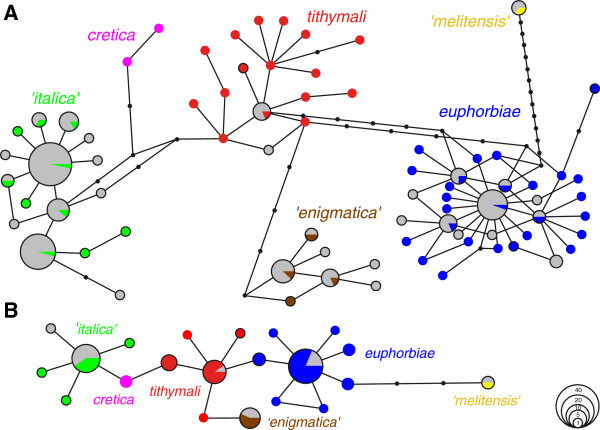
**Haplotype networks for partial mitochondrial COI/II genes sequences of historical specimens from Italy and Malta.** (**A**) Network for contigs of all three fragments (B, H, L) of 143 specimens. (**B**) Network for fragment L of further 20 specimens which did not yield sequences for all three fragments. Included in each network are the 60 haplotypes of recent Mediterranean HEC samples from the data set of Hundsdoerfer *et al.*[31] (reduced to the three fragments used in the present study). Haplotypes without black border did not occur in Italy or Malta. Colour coding of recent samples follows coding of mitochondrial lineages in Hundsdoerfer *et al.*: Figure 3 [31]; historical samples are coloured grey. Black nodes represent one substitution each.

By means of a haplotype network we were able to assign the specimens confidently to a lineage (Figure 1A). Based on fragment L alone, unambiguous lineage assignment was also possible for the 20 specimens for which all three fragments could not be gained (Figure 1B). We detected 16 ‘ghost’ haplotypes (haplotypes that are not present in the recent samples from 2004–2010 but only in the historical samples). Six belonged to the '*italica*' lineage cluster, six to *euphorbiae*, three to '*enigmatica*', and one to the *tithymali* cluster (Figure 1). In contrast, four '*italica*', two *euphorbiae*, one '*enigmatica*' and one of the *tithymali* haplotypes were not found in the historical but only in the recent sampling. We detected misincorporations in 28 out of the 163 specimens that yielded sequences (17.2%) with a mean year of origin at 1929. C→T (41.7%) and G→A (35.4%) transitions were most frequent followed by T→C (10.4%), A→G (6.3%) and single occurrences of A→T, A→C and C→G.

### Lineage distribution through time

**Figure 2:**
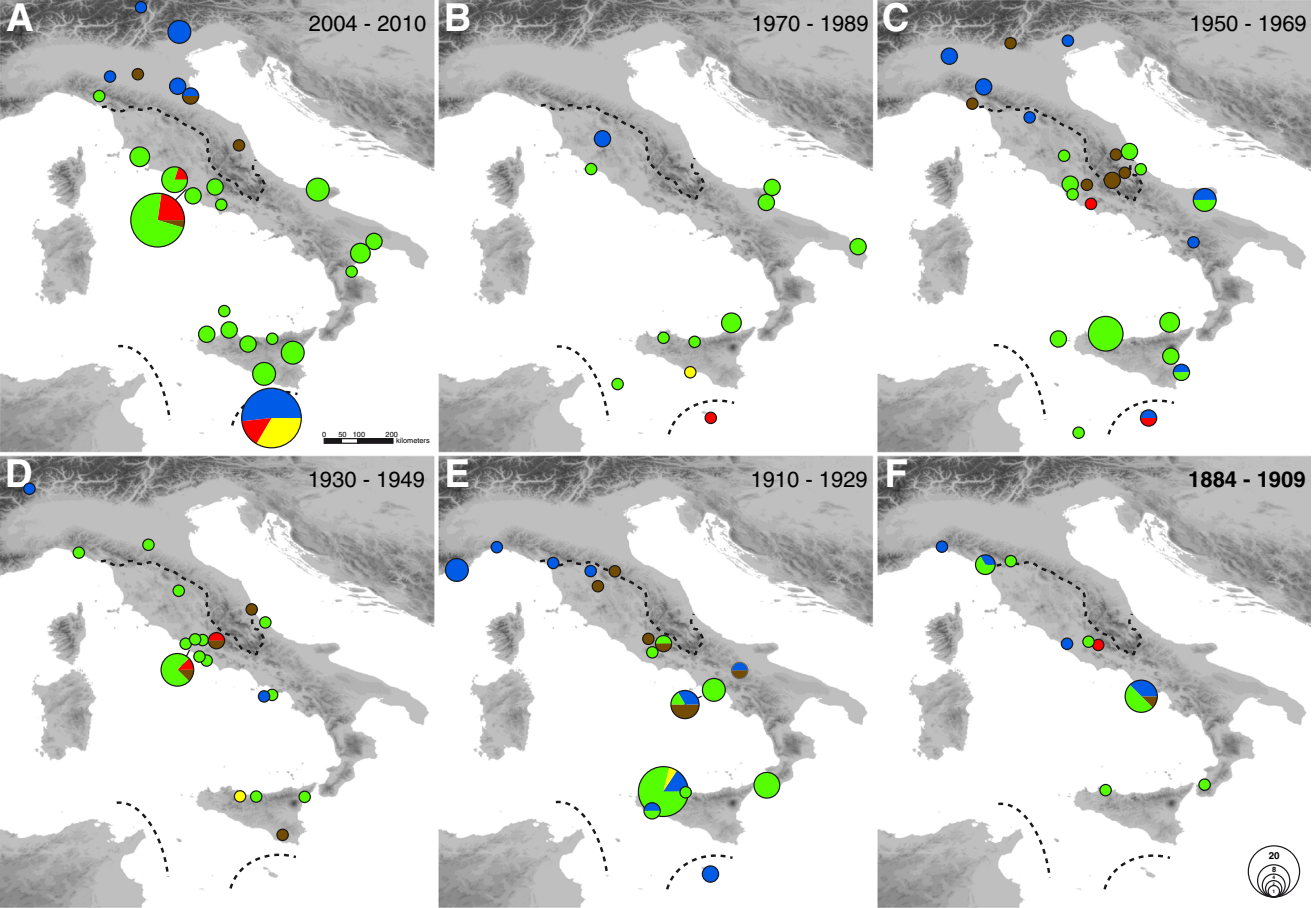
**Distribution of mitochondrial lineages of the HEC in Italy and Malta throughout the past 126 years.** (**A**) Recent distribution in 2004–2010 (data from Hundsdoerfer *et al.*[31]). (**B-F**) Historical samples are pooled to time periods of two decades each. Size of pies corresponds to the number of individuals per locality. Mitochondrial lineages of the HEC are defined according to Hundsdoerfer *et al.*[31] and Figure 1; colours correspond to the lineages (see Figure 1). Dashed black lines mark the border of the '*italica*' core region deduced from the recent distribution in 2004–2010 for use in Figure 4.

**Figure 3:**
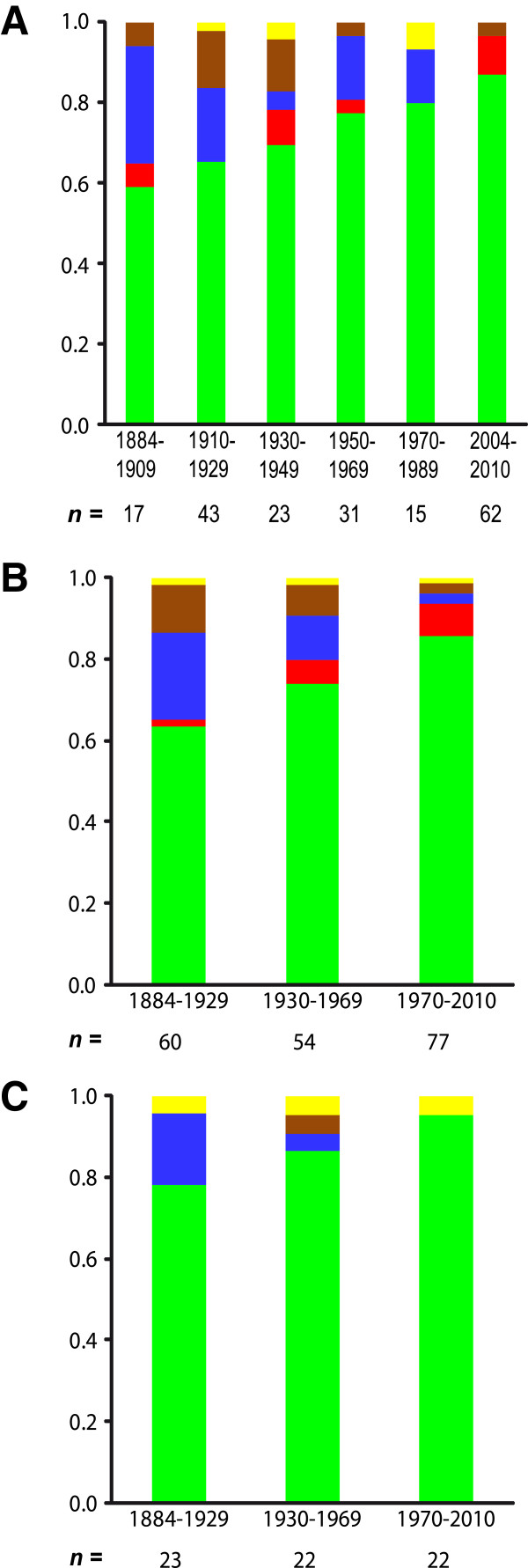
**Composition of mitochondrial lineages in the total HEC population of the extant '*italica*' core region.** (**A**) Population composition of the extant '*italica*' core region (see dashed lines in Figure 2) per time period of two decades each, (**B**) per time period of three decades each. (**C**) Population composition of Sicily per time period of three decades each. Colours correspond to the lineages (see Figure 1). Numbers of individuals (*n*) per time period are given.

Today (Figure 2A, 2004–2010), the '*italica*' lineage dominates - with the exception of a few specimens around Rome - an area that we define as its core region. This area encompasses Sicily and Southern to Central Italy up to the coastal and lower regions of northern Tuscany (see dashed lines in Figure 2). The two European mainland lineages, *euphorbiae* and '*enigmatica*', are absent from this area but replace '*italica*' northeast of the Apennines. Looking back in time, we also find *euphorbiae* and '*enigmatica*' in today’s '*italica*' core region (Figure 2B-F). The last evidence for the *euphorbiae* lineage in this region is from 1970 in Tuscany (Additional file 1: Table S1: #5608, 5609; Figure 2B), from 1968 and 1959 in southern Italy (Additional file 1: Table S1: #7316, 7317, 5580) and from 1955 on Sicily (Additional file 1: Table S1: #7322; Figure 2C). Thus, *euphorbiae* disappeared earlier from the more southern areas. Its former substantial abundance of 29% in the '*italica*' core region’s population in 1884–1909 declined with fluctuations to zero in the recent sampling (Figure 3A). If the samples are pooled into time periods of three decades, the decline is seen to be gradual (Figure 3B). Likewise, *euphorbiae* gradually declined from 17% in 1884–1929 to zero in 1970–2010 in the Sicilian subpopulation (Figure 3C). The '*enigmatica*' lineage inhabits the higher altitudes of the Apennines in Central Italy (Figure 2C) and has also been occasionally present in adjacent areas of Latium at lower altitudes throughout the Twentieth Century until today (Figure 2A/C/E). Furthermore, it also occurred in Southern Italy until at least 1920 (Figure 2E/F; Additional file 1: Table S1: #7269, 7270, 8670, 8676) and on Sicily at least once in 1937 (Figure 2D; Additional file 1: Table S1: #5727). The '*enigmatica*' lineage was most frequent in the '*italica*' core region in 1910–1929, comprising 14% of the total population (Figure 3A). It gradually declined in this region from 12% to 3%, if the samples are pooled into time periods of three decades (Figure 3B). Together, the two European mainland lineages *euphorbiae* and '*enigmatica*' decreased from 35% to 3% over the entire time period of about 120 years considered here (Figure 3A).

In contrast, the proportion of '*italica*' in the total population of its core region steadily increased throughout the Twentieth Century from 59% in 1884–1909 to 87% in 2004–2010 (Figure 3A). The curve is strongly linear (y = 0.111x + 0.519; R^2^ = 0.990). This trend also proves to be robust when we pooled the samples into three decades per time period (Figure 3B) or if only Sicily is considered. The '*italica*' lineage gradually increased its abundance in Sicily from 78% in 1884–1929 to 95% in 1970–2010 (Figure 3C). On the Italian mainland, the North African *tithymali* lineage occurs only in a restricted area around Rome at a rather low frequency throughout at least the past 100 years (Figure 2C/D/F). It slightly increased in abundance in the '*italica*' core region’s population from 2% to 8%, if the samples are pooled into time periods of three decades (Figure 3B). In contrast, we did not detect it in other areas of Italy or on Sicily throughout the late Nineteenth and Twentieth Centuries. The Southern lineages, '*italica*' and the closely related *tithymali*, together increased from 65% to 97% of the '*italica*' core region’s population.

**Figure 4:**
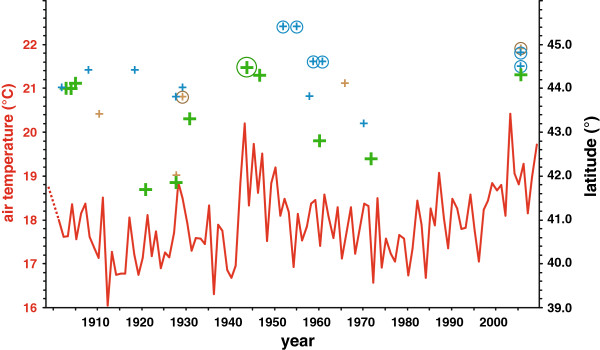
**Northernmost occurrences of '*italica*' haplotypes in northern Central Italy in comparison with mean summer/autumn temperatures.** The red curve represents mean air temperature between June and October per year at an exemplary grid point in NW-Tuscany (44.25°N, 9.75°E); the dashed red line indicates that relatively warm summers preceded the diagrammed data in the 1890s in Italy, see [68,87]. The northernmost '*italica*' occurrences (green crosses) and all occurrences of other lineages in more northern localities (smaller crosses with colours corresponding to the lineages, see Figure 1) are plotted according to date and latitude. Circles surrounding crosses indicate occurrence north of the Apennines.

We were able to gather only few historical specimens of the HEC from Malta, and most of these samples did not yield sequences (see Additional file 1: Table S1). Hence, we cannot deduce historical demographics for the archipelago. However, we were able to show that *euphorbiae* has been present on Malta since at least 1920 (Figure 2E; Additional file 1: Table S1: #5735, 5736) and *tithymali* was already present by 1954 (and also detected later in 1980) (Figure 2C/B; Additional file 1: Table S1: #8649, 8004). In the five sequences obtained, the endemic '*melitensis*' lineage could not be confirmed for Malta in historical times. However, we found it occasionally on the adjacent island of Sicily as single specimens (Figure 2B/D/E; Additional file 1: Table S1: #5432, 6696, 7941), representing a comparatively stable proportion of 1–2% of the entire Italian (Figure 3B) or 4-5% of the Sicilian population (Figure 3C) through time. As in the recent sampling, '*italica*' could not be found on Malta in historical times.

The mean air temperatures for summer/autumn in Italy, which corresponds to the main period of adult flight and larval activity of the HEC, fluctuated throughout the Twentieth Century. Northern Tuscany experienced especially warm summers at the end of the 1920s, in the 1940s and in the first decade of the Twenty-first Century (Figure 4). Correspondingly, '*italica*' occurs in more northern areas in Tuscany and Emilia-Romagna during these warmer periods (Figure 4; see also Figure 2A/D/F). In 1943, the second warmest summer recorded, '*italica*' was even found north of the Apennines near Bologna (Figure 4; also see Figure 2D). Equally, the northernmost specimens of the '*italica*' lineage were found much further south, in southern Tuscany or Latium, in colder periods in the 1910s to 1920s and mid-1950s to 1970s (Figure 4; see also Figure 2B/C/E).

## Discussion

### Lineage distribution through time

Geographic isolation in different refuges during the Ice Ages generally led to the evolution of differentiated genetic lineages in European biota [5,8,32]. Based on the investigation of present day biogeography, these lineages generally did not mix to a greater geographical extent when they repopulated more northern areas in the postglacial but formed confined contact zones across Europe [5,8,32]. By implication, it is often argued that these spatially separated lineages may represent distinct evolutionary entities (subspecies) which remained predominantly separated since their origin and that the suture zones were probably stable since first contact thousands of years ago [8,11]. Likewise, a separate HEC entity in Southern Italy was supported by the prevalence of the mitochondrial '*italica*' lineage (Figure 2A) [31] and distinct larval morphology [25] as well as the frequent occurrence of a reddish adult forewing pattern variety [34-37].

In contrast, our study revealed that the distribution patterns of the HEC’s mitochondrial lineages have been highly dynamic only during the last century and that the pattern of the integral '*italica*' core region evolved very recently (although 120 years translate to about 300 generations assuming two to three generations per year [23,24]). The European mainland lineages, *euphorbiae* and '*enigmatica*' were once mixed in a moderate frequency among '*italica*' throughout the entire range of the latter, thereby connecting the present-day *euphorbiae* exclave on Malta with Central European *euphorbiae* north of the Apennines. Thus, there was no integral source range of a supposed Italian entity a couple of decades ago. Instead, the past situation in Italy resembles the present day sympatric occurrence of *euphorbiae* and '*enigmatica*' in most of the European distribution range of the HEC (see [31]). In the recent sampling, single specimens of the '*italica*' lineage have also been found in southern Greece, north-eastern Spain and Morocco among the dominant *euphorbiae* and '*enigmatica*' or *tithymali* respectively [31] which could have been attributed to recent dispersal and introgression of an isolated Italian entity. However, the observed past admixture of lineages in Italy and thus the absence of a pure source range of '*italica*' strongly favour ancestral polymorphisms with lineage sorting ‘in action’. Concordantly, the frequency of the reddish adult wing pattern variety appears not to support a separate entity as it did not rise in frequency in correlation with '*italica*' in Italy through time but fluctuated independently (data not shown; but see Additional file 1: Table S1).

Environmental factors that probably caused the demographic changes during the last Century (see below) also fluctuated throughout the entire postglacial [39]. In deduction, the biogeographical pattern of the HEC would have changed repeatedly during this period. Thus, mitochondrial lineage compositions in refuges during the last Ice Age and/or even the actual areas of origin of the different lineages in preceding Ice Ages can neither be reliably deduced from the present day nor the historic Nineteenth Century’s biogeography. Given the HEC is a mobile, rather common and widespread species [23,24], it appeared to fulfil the criteria of a good model for assessing biogeographical patterns *sensu* Schmitt [8]. Consequently, a wide range of other species could have likewise experienced such fast demographic changes caused by the same general environmental changes. A similar sorting of mitochondrial lineages into geographically separate areas in an unexpectedly short time period could be shown for the Alaskan brown bear [18], an organism with a very different life history strategy. However, further studies on historic biogeographies of additional taxa are needed to investigate if such a fast lineage sorting is a common phenomenon or if the HEC and the Alaskan brown bear rather represent single cases.

### Causes for demographic changes

The HEC is a strong flier [24,26] and a widespread taxon that has not been considered rare or endangered in Italy [40,41]. HEC caterpillars live in a variety of xerothermic open habitats [23,24], e.g. natural beach dunes, field margins, uncultivated land, *etc.* These habitats were promoted by deforestation and extensive agriculture by early human settlers and thus probably quite abundant in historical times in Italy [42-45]. However, they started to decline with the acceleration of anthropogenic pressure on the landscape about 150–100 years ago [42,46-48] in correlation with the observed lineage sorting. Coastal dune habitats lost about three-quarters of their former extent during the last century in Italy [47,49,50]. In increasing areas of intensive agriculture [46], the use of pesticides had a growing impact on insect populations of the field margins [51-53] while vegetation succession on abandoned land [46,48] also led to population extinctions of many species of open habitats in Italy [44,54]. For instance, the gradual decline in the number of HEC samples from Capri in the natural history collections (see Figure 2) is postulated to reflect a real decline in population size of the HEC rather than a collector bias since former habitats appeared to be largely overgrown on the island nowadays (unpubl. observations by MBM). Most habitats where we found HEC caterpillars in Italy recently were rather small and sometimes temporary, thus many populations probably undergo bottlenecks and/or local extinctions and founder effects. Hence, we postulate that genetic drift promoted the observed lineage sorting process. It would statistically favour the fixation of the already most abundant lineage '*italica*' in Southern Italy as we actually observed in our study. Genetic drift is strongest in small populations [55]. Thus, it is particularly strong for mitochondrial markers because they are haploid and maternally inherited, thus further reducing the effective population size to one-quarter of that of nuclear loci [2,4]. Furthermore, the existence of biogeographical patterns based on maternally inherited mitochondrial markers [31] supports the hypothesis that females are rather faithful to a habitat [1]. Thus, mitochondrial gene flow between populations is likely to have decreased with increased habitat fragmentation [56]. In contrast to the majority of small habitats, genetic drift would not play a major role in large, temporally stable populations, such as the one near Rome airport (Figure 2A: large pie in Central Italy) or on Malta. Consequently, the mitochondrial polymorphism could be maintained there. Nevertheless, overall gene flow between the fragmented habitats of the HEC is likely to be maintained more strongly by dispersing males, as postulated by Hundsdoerfer *et al.*[29]. Further investigations using nuclear microsatellite markers [57,58] are promising to elucidate the overall gene flow and the actual extent of current biological entities.

In addition to genetic drift, changes in climatic conditions likely have contributed to the observed demographic changes since the HEC appears to be sensitive to temperature as has been shown for many other lepidopterans [59-61]. The northernmost occurrences of '*italica*' have - though based on only a few specimens - closely followed the fluctuations of mean summer/autumn air temperatures (Figure 4). Similarly fast range extensions in the 1940s and subsequent retractions in the 1950s have also been found in some butterfly species in Britain [59]. Beyond these short term fluctuations, many insect species gradually expanded their ranges in latitude and/or altitude into previously unsuitable areas throughout the Twentieth Century [54,59-64]. For a few insects so far, demographic shifts of molecular markers within the existing range could also be shown in correlation with climate warming [65-67]. Likewise, the observed gradual increase of '*italica*' haplotypes in its core region (Figure 3) correlates with the trend of temperature anomaly values increasing by about 1°C per century over the last 140 years in Italy [68]. In addition, haplotypes of *euphorbiae* disappeared slightly earlier in more southern regions. The suture zone along the Apennines that constitutes the present day northern limit of '*italica*' is remarkably concordant with the northern limit of summer drought [43] and the border between the Mediterranean and the Continental biogeographical region [69]. Furthermore, a climate niche modelling based on the extant distribution of mitochondrial lineages predicted a very congruent northern limit of '*italica*', while *euphorbiae* could potentially be present throughout Italy [31]. Thus, '*italica*' populations appear to be more successful in a Mediterranean climate and outcompeted *euphorbiae* in Southern Italy while they could not establish themselves in the continental climate of the Po Plains yet. A mitochondrial lineage could be indirectly linked to nuclear genes under selection at population level, i.e. individuals of more southerly populations in Italy have a higher probability to bear an '*italica*' haplotype and are also potentially better adapted to a warmer climate based on alleles of their nuclear genome (e.g. tend to undergo an additional generation in autumn before diapause [23,24]). Thus, as the climate warms up, the '*italica*' lineage would concomitantly increase in frequency. Alternatively, it cannot be ruled out that sequence differences in the mitochondrial genes have a direct influence on individual fitness with regard to climatic conditions, since in poikilotherms, the external temperature is directly experienced by the mitochondria [2]. Nevertheless, such adaptations to local climatic conditions are known to occur within widely distributed lepidopteran taxa [70] and thus do not necessarily support a distinct Italian entity.

## Conclusions

The results of our study are in contrast to the common point of view that the pattern of currently geographically separated lineages indicate taxa which have been long term separated since the origin of these lineages in glacial refuges and which did not mix to a greater extent in the current interglacial. Even though it still has to be investigated on further taxa if the fast lineage sorting observed in the HEC during the Twentieth Century is rather a single case or a common pattern, we strongly emphasize that caution should be taken in interpreting modern day biogeographical patterns based on mitochondrial DNA (even if they are supported by congruent morphological patterns) as unaltered in the long term and for drawing hasty taxonomic conclusions based upon them.

## Methods

### Sampling

We applied for a sampling permit of *Hyles euphorbiae* tissue in 27 natural history collections, covering the museums most relevant to the study area (Table 1), to balance a local collectors bias as much as possible [12]. The target area for the moths’ origin was confined to Italy south of the Po River and Malta. We only used specimens with complete data (date and locality) or where these data could be reliably inferred e.g. from the collectors name (Additional file 1: Table S1). From large series of specimens collected on the same date and in the same locality, we sampled only a subset of the most different morphs to minimize the chance of sampling siblings. We plucked one to three legs per individual except for the specimens from the Natural History Museum, London (NHM). From these samples tissue was taken from the abdomens to minimize damage to the specimens (see below).

### Laboratory techniques

We conducted the laboratory work in a dedicated ancient DNA clean-laboratory with Class II Safety Cabinets (HERAsafe KSP9; Thermo Scientific, Waltham, USA) and 4 hours of UV-light decontamination between the working steps. Tissue sampling of abdomens followed the protocol of Hundsdoerfer & Kitching [71] whereby abdomens are broken off carefully, macerated and glued on again after drying. DNA was isolated with the AGOWA sbeadex Forensic Kit (AGOWA Genomics, Berlin, Germany) using 120 μL of macerate or one leg cut into small pieces. We modified the manufacturer’s protocol by digestion overnight and using only 60 μL elution buffer. To avoid cross contaminations (especially when processing macerates), we spun down the liquid inside the vials after every mixing step, used a fresh piece of absorbent paper to open each vial and decontaminated gloves with DNA away (Carl Roth, Karlsruhe, Germany) after processing each sample.

The most informative fragments of marker genes were chosen for enzymatic amplification (PCR). The definition of the mitochondrial lineages of the HEC is based upon sequences of the genes for CO I/II and the interposed gene for t-RNA leucine (2284 bp in total; [34]). Specific primers to sequence these genes from historical specimens in 13 overlapping fragments (fragments A to M) had already been designed for the entire genus *Hyles*, see [71]. In the present study, we used a subset of three fragments covering 794 bp in total (fragment B: 277 bp, H: 280 bp, L: 237 bp). One primer had been published previously; the others were designed specifically for *Hyles* or the HEC (see below). They were chosen to cover parsimony-informative sites representing autapomorphies of each of the six known lineages of the HEC in the Mediterranean region (Additional file 2: Table S2; only the *tithymali* lineage does not have autapomorphic PI sites in the complete sequence of all three genes). Except for fragment B, where *tithymali* and '*enigmatica*' have identical sequences, each lineage can be discriminated by every fragment alone (Additional file 2: Table S2).

We performed a multiplex PCR setup [72] to amplify all three fragments at once in a separate Class II Safety Cabinet (Thermo Scientific) in the dedicated clean-laboratory using the “Type-it Microsatellite PCR Kit” (Qiagen, Hilden, Germany). Amplification was carried out in a total volume of 20 μL containing 1x Type-it Multiplex PCR Master Mix (Qiagen), 0.2 μM of each primer (biomers.net, Ulm, Germany; fragment B: "*HylesCOIca100f*": 5^′^-TAAGATTAYTAATTCGAGCAG-3^′^, "*MLepR1*": 5^′^-CCTGTTCCAGCTCCATTTTC-3^′^[73]; H: "*HEC-COIca1110f*" 5^′^-ATGATACATATTATGTTGTAGC-3^′^, "*HEC-COIca1350r*" 5^′^-GARATATATGAYCCTAATGATGA-3^′^; L: "*HylesMCOIIf*" 5^′^-GATAYTGAAGATATGAATATTC-3^′^, "*HylesCOIIca2125r*" 5^′^-TTGTTTGRTTTAAACGTCCAGG-3^′^) and 5 μL of DNA extract. The PCR comprised denaturation at 95°C for 5 min, 35 cycles of 95°C for 30 s, 57°C for 1 min 30 s and 72°C for 45 s and final elongation at 60°C for 30 min and was conducted on an Eppendorf Mastercycler gradient or gradient pro S (Eppendorf, Hamburg, Germany) in a separate laboratory building.

A subsequent simplex PCR was set up under a “UV Sterilizing PCR Workstation” (peqLab Biotechnologie, Erlangen, Germany) with a total volume of 20 μL containing 1 unit *Taq* polymerase (Bioron, Ludwigshafen, Germany), 1x ‘complete’ PCR buffer (Bioron), 0.2 mM of each dNTP (Fermentas, St. Leon-Rot, Germany), 0.25 μM each of forward and reverse primer for one fragment and 1 μL of the multiplex PCR product. The PCR comprised denaturation at 94°C for 4 min followed by 40 cycles of 94°C for 45 s, 47°C for 45 s and 72°C for 45 s and final elongation at 72°C for 10 min.

Success of the simplex PCR was visualized on a 2% Agarose gel stained with GelRed (Biotium Inc., Hayward, USA). Only products with a visible band of the fragment were used for the following cycle sequencing reaction. The PCR product was purified with the Exo SAP-IT enzymatic cleanup (USB Europe GmbH, Staufen, Germany) at 37°C for 30 min and 80°C for 15 min. 1 μL of the product was used for cycle sequencing with the BigDye Terminator v. 3.1 Cycle Sequencing Kit (Applied Biosystems, Life Technologies Corporation, Carlsbad, USA) following the manufacturer’s instructions. The cycle sequencing product was purified using Sephadex G-50 fine (GE Healthcare, München, Germany) and sequenced in both directions on an ABI 3130xl sequencer (Applied Biosystems).

### Authentication

To monitor contaminations, we followed the common recommendations [11,13,72] to include negative controls in DNA extraction and add further blank samples to each of the subsequent PCRs. Furthermore, the three different fragments could be used to detect contaminations as chimeras (i.e. contradicting lineage assignment between different fragments of the same specimen). As a positive control [74-76], we included DNA from a fresh larval sample of the distantly related *Hyles livornica* (MTD-# 7528; ~30 ng DNA per reaction), which is clearly distinguishable from the target sequences in every fragment (Additional file 2: Table S2). This DNA was stored in the PCR laboratory and added immediately before starting the multiplex-PCR to detect contamination effects of neighbouring vials and to monitor PCR success.

For validation, DNA extraction and amplifications were processed a second time for some individuals that belonged to a lineage that was unexpected in its locality (see Additional file 1: Table S1). Most of the macerate samples were also processed twice. We thereby achieved six independent amplifications (twice three fragments) for crucial specimens and thus met the recommendations for repeated extractions and amplifications in ancient DNA studies [11]. Nevertheless, repeated extraction was not possible for all specimens since sometimes only one leg had been plucked (common among studies on historical insect specimens: [17,19,22]). In a few cases, we were able to amplify only the shortest fragment L from a specimen. However, since unambiguous lineage assignment was still possible by means of this fragment (Additional file 2: Table S2; Figure 1B) we included these specimens in further statistical analyses. Most individuals possessing a potential ‘ghost haplotype’ (i.e. a substitution or ambiguity at an invariable site of the extant samples data set) were processed again from multiplex PCR onwards to verify these or detect and correct nucleotide misincorporations [77,78].

We confirmed that the different lineages of the HEC are derived from transcribed mitochondrial genes rather than nuclear pseudogenes ('numts') by performing a cDNA transcription of mRNA from frozen tissue of recently caught HEC specimens (Mende *et al*., in prep.). Specimens of the lineages *euphorbiae*, '*melitensis*', '*italica*' and '*enigmatica*' were included, whereas specimens of the lineages *tithymali* and *cretica* were not available as RNA containing tissue.

### Data analysis

We calculated a Spearman’s rank correlation between PCR success of the samples and sample ages and/or latitude of the storage place with the ‘stats’ package in R 2.14.0 [79]. The latitude was used as a variable for the mean temperature to which the specimens were exposed, because temperatures are generally higher outside and likewise inside the collection’s building in more southern cities (open windows, nor air-conditioning; unpubl. observations by MBM). However, the potential influence of different preparation methods [80] could not be addressed.

We aligned the sequences and assembled the fragments per individual with BioEdit 7.0.9.0 [81]. The alignment is available in the Dryad digital repository at doi:10.5061/dryad.1g98b. Mutational relationships of the contigs of all three fragments per individual were examined by a median joining network analysis in Network 4.5.1.0 (Fluxus Technology, Suffolk, UK). In addition to the historical samples, the network contains the 60 haplotypes of recent Mediterranean HEC samples from the reduced data set of Hundsdoerfer *et al.*[31] (only the three fragments used in the present study; ambiguities in these sequences were corrected according to the sole or most common nucleotide for the lineage at that position). We also calculated networks for individuals, of which only sequences of two fragments (B, L or H, L, not shown) or just the shortest fragment (L) could be obtained, again including the 60 recent sample sequences.

For illustration of the geographical distribution of mitochondrial lineages, we pooled the historical samples to five time periods of about two decades each. We plotted the samples as pie charts on a map for each time period using ArcGIS 10 (Esri Inc., Redlands, USA). The topographic map was generated using the SRTM 90 m Digital Elevation Dataset 4.1 [82]. For comparison with the extant distribution of lineages we used the data from Hundsdoerfer *et al.*[31] of 62 specimens collected in Italy and Malta between 2004 and 2010 (Figure 2A). We calculated bar charts to illustrate the proportion of every lineage in the total population of the extant '*italica*' core region (see Results for definition) or only Sicily (as the most pure '*italica*' population without interference from the suture zone in Northern Italy). Samples were pooled to time periods of two or three decades each to obtain statistically relevant numbers per period. Geographic and chronologic pooling of specimens is mostly unavoidable in historic DNA studies (also see [83]) but appears reasonable for our purpose since the HEC is known to be a strong flier [24,26].

To compare the northernmost occurrences of '*italica*' haplotypes in northern Central Italy with mean summer/autumn air temperatures we used a graph of CRU temperature data from 1901–2009 [84,85] (improved by [86]) for the grid point 44.25 °N, 9.75 °E. This point is closest to the northernmost occurrence of '*italica*' in the recent sampling (Fillatiera, NW-Tuscany). Summer/autumn refers to June - October, the main period of adult flight and larval activity of the HEC in Italy. According to their latitude, we plotted the northernmost '*italica*' occurrences and all other more northern occurrences (localities from northern Lazio to the Po River plains) onto the graph.

## Competing interests

The authors declare that they have no competing interests.

## Author’s contributions

AKH had the initial idea and together with MBM designed the study. MBM sampled the specimens, carried out the experimental work, analyzed the data and wrote the manuscript. AKH contributed to the analysis of climate data, the discussion and manuscript editing. Both authors have approved of the final version of the manuscript.

## Supplementary Material

Additional file 1: Table S1 Origin and other information about the historical samples included in this study. *MTD-#*: voucher number in the invertebrate tissue catalogue of the Museum für Tierkunde, Dresden; *Museum*: source of the sample (abbreviation according to Table 1); *Locality*: data in square brackets completed by inference; *Coordinates*: inferred from locality data on label, italic coordinates indicate imprecise locality; *Mt-lineage*: mitochondrial lineage according to Hundsdoerfer *et al.*[31], ^BL^ = no successful PCR of fragment H, ^HL^ = no successful PCR of fragment B, ^L^ = successful PCR of fragment L only, --- = no successful PCR of any fragment; *Extr*: 2x = repeated DNA extraction; *Variety*: *grentzenbergi* = reddish forewing pattern variety. Click here for file

Additional file 2: Table S2 Variable sites of the three amplicons used in this study for the Mediterranean lineages of the HEC. Consensus sequences for each Mediterranean HEC lineage of all individuals from Hundsdoerfer *et al*. [31] were reduced to variable sites of the three fragments B, H, L. Character states for the positive control outgroup *H. livornica* are reported for comparison. Positions are numbered according to the 2284 bp alignment of COI/II genes of Hundsdoerfer *et al*. [31]; fragment B: position 89–365, fragment H: 1094–1373, fragment L: 1909–2145. Round brackets indicate that not all individuals of the lineage bear the substitution; lowercases indicate rare substitutions in only few or single specimens. Click here for file
