# Supplementary material for: Mitochondrial lineage sorting in action – historical biogeography of the Hyles euphorbiae complex (Sphingidae, Lepidoptera) in Italy
Source: BMC Evol Biol. 2013 Apr 18;13:83. doi: 10.1186/1471-2148-13-83 (PMC3655913; doi:10.1186/1471-2148-13-83)
Supplement: Additional file 1: Table S1 — Origin and other information about the historical samples included in this study. MTD-#: voucher number in the invertebrate tissue catalogue of the Museum für Tierkunde, Dresden; Museum: source of the sample (abbreviation according to Table 1); Locality: data in square brackets completed by inference; Coordinates: inferred from locality data on label, italic coordinates indicate imprecise locality; Mt-lineage: mitochondrial lineage according to Hundsdoerfer et al.[31], BL = no successful PCR of fragment H, HL = no successful PCR of fragment B, L = successful PCR of fragment L only, --- = no successful PCR of any fragment; Extr: 2x = repeated DNA extraction; Variety: grentzenbergi = reddish forewing pattern variety. [file 1471-2148-13-83-S1.doc]

| **MTD-#** | **Museum** | **Locality** | **Coordinates** | **Date; collector/collection** | **Mt-lineage** | **Extr.** | **Variety** |
| --- | --- | --- | --- | --- | --- | --- | --- |
| 5388 | ZMH, Hamburg | [Italy, Lazio,] Ostia | 41,7 N, 12,3 E | 07.08.1938 e.l. | '*italica*' |  | *grentzenbergi* |
| 5389 | ZMH, Hamburg | [Italy, Lazio,] Ostia | 41,7 N, 12,3 E | 17.08.1938 e.l. | *tithymali* | 2x | *grentzenbergi* |
| 5390 | ZMH, Hamburg | [Italy, Lazio,] Ostia | 41,7 N, 12,3 E | 10.07.1938 e.l. | '*italica*' |  | *grentzenbergi* |
| 5391 | ZMH, Hamburg | [Italy, Lazio,] Ostia | 41,7 N, 12,3 E | 21.07.1938 e.l. | '*italica*' |  | *grentzenbergi* |
| 5392 | ZMH, Hamburg | [Italy, Lazio,] Ostia | 41,7 N, 12,3 E | 01.08.1938 e.l. | '*enigmatica*' | 2x |  |
| 5393 | ZMH, Hamburg | [Italy, Lazio,] Ostia | 41,7 N, 12,3 E | 09.07.1938 e.l. | '*italica*' |  |  |
| 5394 | ZMH, Hamburg | [Italy, Lazio,] Ostia | 41,7 N, 12,3 E | 11.08.1938 e.l. | '*italica*' |  |  |
| 5395 | ZMH, Hamburg | [Italy, Sicilia,] Palermo | 38,1 N, 13,3 E | [<1950] Eingang 1960; Dr. H. Bytinski-Salz | '*italica*' ^BL^ |  | *grentzenbergi* |
| 5397 | SMTD, Dresden | [Italy, Campania,] Capri | 40,6 N, 14,2 E | [<1940] Ankauf 1942; Dr. P. Denso | *euphorbiae* |  | *grentzenbergi* |
| 5398 | SMTD, Dresden | [Italy, Sicilia,] Taormina | 37,9 N, 15,3 E | [<1950] Ankauf 1950; W. Heinitz | '*italica*' |  | *grentzenbergi* |
| 5403 | MNB, Berlin | [Italy, Campania,] Capri | 40,6 N, 14,2 E | 27/9/[18]84; Staudinger | '*italica*' |  | *grentzenbergi* |
| 5404 | MNB, Berlin | [Italy, Campania,] Capri | 40,6 N, 14,2 E | 9/9/[18]84; Staudinger | '*italica*' |  | *grentzenbergi* |
| 5405 | MNB, Berlin | [Italy, Campania,] Capri | 40,6 N, 14,2 E | 15/9/[18]84; Staudinger | '*italica*' |  | *grentzenbergi* |
| 5406 | MNB, Berlin | [Italy,] Sicilia, Palermo | 38,1 N, 13,3 E | e.l. 7.9.[18]85; Dr. Struve, Leipzig | '*italica*' |  | *grentzenbergi* |
| 5431 | MSNG, Genova | [Italy,] Sicilia, Isola di Pantelleria | 36,8 N, 12,0 E | 5.X.1986; [Coll. Grillo] | '*italica*' |  | *grentzenbergi* |
| 5432 | MSNG, Genova | [Italy,] Sicilia, Licata | 37,1 N, 13,9 E | 7.VI.1989 ex larva schiusa; [Coll. Grillo] | '*melitensis*' | 2x |  |
| 5433 | MSNG, Genova | [Italy,] Sicilia, Monreale, Giacalone, 700m | 38,0 N, 13,2 E | 20.VIII.1984; [Coll. Grillo] | '*italica*' |  |  |
| 5434 | MSNG, Genova | [Italy,] Sicilia, Isnello, Portella Colla, 1421m | 37,9 N, 14,0 E | 31.VIII.1973; [Coll. Grillo] | '*italica*' |  |  |
| 5435 | MRST, Terrasini | [Italy, Sicilia,] Palermo | 38,1 N, 13,3 E | XII.1928 ex larva; G. Marte; [Coll.] Mariani | --- |  | *grentzenbergi* |
| 5436 | MRST, Terrasini | [Italy,] Lazio, (Nettuno) | 41,5 N, 12,7 E | Prest. 9.[1]939; [Coll.] Mariani | '*italica*' |  |  |
| 5437 | MRST, Terrasini | [Italy, Sicilia] Ustica | 38,7 N, 13,2 E | 23.09.1960; [Coll.] De Stefani | --- |  |  |
| 5438 | MRST, Terrasini | [Italy,] (Sicily), Sferracav.[allo] | 38,2 N, 13,3 E | 19.05.1960; [Coll.] De Stefani | '*italica*' ^BL^ |  | *grentzenbergi* |
| 5576 | MCVR, Verona | [Italy,] Marche, Colle S Marco | 42,8 N, 13,6 E | 7.VI.1966; leg. S. Zangheri | '*enigmatica*' |  |  |
| 5577 | MCVR, Verona | [Italy,] Sicilia, Marettimo | 38,0 N, 12,1 E | 6.V.[19]68; [Coll.] Sergio Zangheri | '*italica*' |  |  |
| 5578 | MCVR, Verona | [Italy,] Sicilia, Marettimo | 38,0 N, 12,1 E | 6.V.[19]68; [Coll.] Sergio Zangheri | '*italica*' |  |  |
| 5579 | MCVR, Verona | [Italy, Emilia-]Romagna, Forli | 44,2 N, 12,0 E | 20.VI.1941; leg. S. Zangheri | --- |  |  |
| 5580 | MCVR, Verona | [Italy,] Lucana [= Basilicata], Nova Sizile | *40,5 N, 15,6 E* | 07-IX-[19]59; leg. S. Zangheri | *euphorbiae* | 2x |  |
| 5581 | MCVR, Verona | [Italy, Veneto,] Ferrara 850/1100m (Mte Baldo) | 45,7 N, 10,8 E | E.8.1967; leg. J. Wolfsberger | '*enigmatica*' |  |  |
| 5582 | MCZ, Roma | [Italy, Toscana,] Vallombrosa | 43,7 N, 11,6 E | 5.8.[1]900; ex coll. Rostagno | --- |  |  |
| 5583 | MCZ, Roma | [Italy, Toscana,] Cortona, 300m | 43,3 N, 12,0 E | 9-[1]931; ex coll. Garavaglia | '*italica*' |  |  |
| 5584 | MCZ, Roma | [Italy, Umbria,] Orvieto, S. Faustino | 42,8 N, 12,2 E | 20.08.1960; Prola [leg.] | '*italica*' |  |  |
| 5585 | MCZ, Roma | [Italy, Umbria,] Orvieto, S. Faustino | 42,8 N, 12,2 E | 31.08.1944; Prola [leg.] | --- |  |  |
| 5586 | MCZ, Roma | [Italy, Lazio,] A Poli, N Roma | 41,9 N, 12,9 E | 24-7-[1]902; ex coll. Rostagno | --- |  |  |
| 5587 | MCZ, Roma | [Italy, Lazio,] a. Poli | 41,9 N, 12,9 E | 26/6/[1]901; ex coll. Rostagno | --- |  |  |
| 5588 | MCZ, Roma | [Italy,] Lazio, Frascoli | *41,7 N, 12,9 E* | 18.IX[1]921; Luigioni [leg.] | '*italica*' ^L^ |  | *grentzenbergi* |
| 5589 | MCZ, Roma | [Italy,] Lazio, Tivoli | 42,0 N, 12,8 E | VI.[1]928; leg. Luigioni | '*enigmatica*' |  |  |
| 5590 | MCZ, Roma | [Italy,] Lazio, Anticoli Corrado | 42,0 N, 13,0 E | 06.10.1937; Prola [leg.] | *tithymali* | 2x | *grentzenbergi* |
| 5591 | MCZ, Roma | [Italy,] Lazio, Anticoli Corrado | 42,0 N, 13,0 E | 07.09.1940; Prola [leg.] | '*enigmatica*' | 2x |  |
| 5592 | MCZ, Roma | [Italy, Lazio,] Olgiata, Roma | 42,0 N, 12,4 E | 10.IX.1968; coll. P. Provera | '*italica*' |  |  |
| 5593 | MCZ, Roma | Italia, Puglia, Lecce, S. Cataldo | 40,4 N, 18,3 E | 10.06.1982; Prola [leg.] | '*italica*' |  |  |
| 5594 | MCZ, Roma | Italia, Puglia, Lecce, S. Cataldo | 40,4 N, 18,3 E | 20.06.1984; Prola [leg.] | '*italica*' |  |  |
| 5595 | MCZ, Roma | [Italy, Sicilia,] Isole Pelagie, Lampedusa | 35,5 N, 12,6 E | 25.05.1956; Prola [leg.] | '*italica*' | 2x |  |
| 5596 | MCZ, Roma | [Italy,] Lazio, Palidoro | 41,9 N, 12,2 E | 25.07.1938; Prola [leg.] | --- |  | *grentzenbergi* |
| 5597 | MCZ, Roma | [Italy,] Lazio, Palidoro | 41,9 N, 12,2 E | 08.06.1938; Prola [leg.] | '*italica*' |  | *grentzenbergi* |
| 5598 | MCZ, Roma | [Italy,] Lazio, Ardea | 41,6 N, 12,5 E | 24.06.1938; Prola [leg.] | '*italica*' |  |  |
| 5599 | MZUF, Firenze | [Italy, Toscana,] Forte dei Marmi | 44,0 N, 10,2 E | 22 Giugno [1]903 ex larvae | --- |  |  |
| 5600 | MZUF, Firenze | [Italy, Toscana,] Forte dei Marmi | 44,0 N, 10,2 E | Maggio[19]04 ex larvae | '*italica*' ^HL^ |  |  |
| 5601 | MZUF, Firenze | [Italy, Toscana,] Forte dei Marmi | 44,0 N, 10,2 E | 30 Genn. [1]903 ex larvae (forzato) | '*italica*' ^HL^ |  |  |
| 5602 | MZUF, Firenze | [Italy, Toscana,] Firenze, Pian di Mugnone | 43,8 N, 11,3 E | 19.IX.[19]56; R.Verity [leg.] | *euphorbiae* | 2x |  |
| 5603 | MZUF, Firenze | [Italia, Toscana,] Migliorini, Pistoiya | 44,0 N, 10,8 E | 18 Marzo 1904 | --- |  | *grentzenbergi* |
| 5604 | MZUF, Firenze | [Italy, Toscana,] Forte dei Marmi | 44,0 N, 10,2 E | le Guigno [1]901 ex larvae | --- |  |  |
| 5605 | MZUF, Firenze | [Italy,] (Toscana), Forte dei Marmi | 44,0 N, 10,2 E | 9 Settembre 1902; [Coll.] R. Verity | *euphorbiae* |  |  |
| 5606 | MZUF, Firenze | [Italy, Toscana,] Camaiore, (Lucchese) | 43,9 N, 10,3 E | Agosto [1]902 | --- |  |  |
| 5607 | MZUF, Firenze | [Italy, Toscana,] Brolio, (Siena) | 43,4 N, 11,5 E | 1911; coll. Batt. Ricasoli; R. Verity; var. *etrusca* nova | '*enigmatica*' ^L^ |  |  |
| 5608 | MZUF, Firenze | [Italy, Toscana,] Asciano | 43,2 N, 11,6 E | 1970 | *euphorbiae* ^BL^ |  |  |
| 5609 | MZUF, Firenze | [Italy, Toscana,] Asciano | 43,2 N, 11,6 E | 1970 | *euphorbiae* |  | *grentzenbergi* |
| 5610 | MSNTUP, Pisa | [Italy, Toscana,] Ansedonia | 42,4 N, 11,3 E | [~1972]; F. Strumia [leg.] | --- |  |  |
| 5611 | MSNTUP, Pisa | [Italy, Toscana,] Ansedonia | 42,4 N, 11,3 E | [~1972]; F. Strumia [leg.] | '*italica*' |  |  |
| 5727 | SMF, Frankfurt | [Italy, Sicilia,] Ragusa | 36,9 N, 14,7 E | 13.9.1937; Prier | '*enigmatica*' | 2x |  |
| 5731 | SMF, Frankfurt | [Italy, Campania,] Ins. Capri | 40,6 N, 14,2 E | 1888 | *euphorbiae* | 2x | *grentzenbergi* |
| 5735 | SMF, Frankfurt | Malta, (Citadelle) | 35,9 N, 14,4 E | 1920, Pleimes | *euphorbiae* | 2x |  |
| 5736 | SMF, Frankfurt | Malta, (Citadelle) | 35,9 N, 14,4 E | 1920, Pleimes | *euphorbiae* |  |  |
| 5737 | SMF, Frankfurt | [Italy, Calabria,] mer. Aspromonte, 900m | 38,2 N, 15,9 E | e.l. 25.IX.1920; H. Stauder legit | '*italica*' |  | *grentzenbergi* |
| 5743 | SMF, Frankfurt | [Italy,] Calabria, Aspromonte | 38,2 N, 15,9 E | VII.1914; H. Stauder [leg.] | '*italica*' |  |  |
| 5744 | MSNG, Genova | [Italy,] Liguria, Genova, Palmaro | 44,4 N, 8,8 E | VIII.1919 | *euphorbiae* |  |  |
| 5745 | MSNG, Genova | [Italy,] Liguria, Lavagna, Cavi | 44,3 N, 9,4 E | V.1947 | '*italica*' | 2x |  |
| 5746 | MSNG, Genova | [Italy,] Liguria, Capo Spezia | 44,1 N, 9,8 E | VI.1966 | '*enigmatica*' |  |  |
| 5747 | MSNG, Genova | [Italy,] Toscana, Isola del Giglio | 42,4 N, 10,9 E | VII.1901 | --- |  |  |
| 5748 | MSNG, Genova | [Italy,] Abruzzo, Pescara | 42,5 N, 14,2 E | 1.VI.1947; [Coll. Barbera] | '*italica*' |  |  |
| 5749 | MSNG, Genova | [Italy,] Abruzzo, Pescara, Pineta | 42,5 N, 14,2 E | 18.V.1950; [Coll. Barbera] | '*italica*' |  |  |
| 5750 | MSNG, Genova | [Italy,] Abruzzo, Ovindoli, San Potito, 1050m | 42,1 N, 13,5 E | 10.VIII.1950; [Coll. Barbera] | '*enigmatica*' |  |  |
| 5751 | MSNG, Genova | [Italy,] Abruzzo, Ovindoli, San Potito, 1050m | 42,1 N, 13,5 E | 21.VII.1955; [Coll. Barbera] | '*enigmatica*' |  |  |
| 5752 | MSNG, Genova | [Italy,] Lazio, Roma | 41,9 N, 12,3 E | 14.IX.1961; [Coll. Barbera] | '*italica*' |  |  |
| 5753 | MSNG, Genova | [Italy,] Lazio, Roma, zona Marcigliana | 42,0 N, 12,6 E | 28.VIII.1949; [Coll. Barbera] | '*italica*' |  |  |
| 5754 | MSNG, Genova | [Italy,] Lazio, dint. di Roma, Monte Sacro | 41,9 N, 12,5 E | 31.VIII.1937; [Coll. Barbera] | '*italica*' |  |  |
| 5755 | MSNG, Genova | [Italy,] Lazio, dint. di Roma, Acilia | 41,8 N, 12,4 E | 7.V.1957; [Coll. Barbera] | '*italica*' |  |  |
| 5756 | MSNG, Genova | [Italy,] Lazio, Ostia Lido | 41,7 N, 12,3 E | IX.1938; [Coll. Barbera] | '*italica*' |  |  |
| 5757 | MCSNM, Milano | [Italy,] Lombardia, Milano | 45,4 N, 9,2 E | 09.09.[19]55 | *euphorbiae* |  |  |
| 5758 | MCSNM, Milano | [Italy,] Veneto, Treviso, Ponte della Priula | 45,8 N, 12,3 E | 29.08.[19]58 | *euphorbiae* |  |  |
| 5759 | MCSNM, Milano | [Italy,] Emilia Romagna, Varano de' Melegari | 44,7 N, 10,0 E | 08.[19]25 | --- |  |  |
| 5760 | MCSNM, Milano | [Italy,] Toscana, Firenze | 43,8 N, 11,3 E | 09.[19]28 | *euphorbiae* | 2x |  |
| 5761 | MCSNM, Milano | [Italy,] Toscana, Ronta | 44,0 N, 11,4 E | 06.[19]26 | --- | 2x |  |
| 5762 | MCSNM, Milano | [Italy,] Toscana, Alpi Apuane, Foce di Mosceta | *44,0 N, 10,3 E* | 07.[19]29 | *euphorbiae* |  |  |
| 5763 | MCSNM, Milano | [Italy,] Lazio, Latina | 41,5 N, 12,9 E | 02.-04.09.[19]51 | *tithymali* | 2x | *grentzenbergi* |
| 5764 | MCSNM, Milano | [Italy,] Lazio, Tivoli | 42,0 N, 12,8 E | 08.09.[19]54 | '*enigmatica*' |  |  |
| 5765 | MCSNM, Milano | [Italy,] Piemonte, Macugnaga | 46,0 N, 8,0 E | e.l. 13.08.[19]38; [Coll. Pozzi] | *euphorbiae* |  |  |
| 5766 | MCSNM, Milano | [Italy,] Emilia Romagna,Calestano | 44,6 N, 10,1 E | e.l. 06.[19]59; [Coll. Pozzi] | *euphorbiae* |  |  |
| 5767 | MCSNM, Milano | [Italy,] Emilia Romagna,Calestano | 44,6 N, 10,1 E | e.l. 07.[19]61; [Coll. Pozzi] | *euphorbiae* |  |  |
| 5768 | MCSNM, Milano | [Italy,] Abruzzo, Capo di Monte | *42,3 N, 13,8 E* | 16.07.[19]69; [Coll. Pozzi] | '*enigmatica*' |  |  |
| 5769 | MCSNM, Milano | [Italy,] Emilia Romagna, Casinalbo | 44,6 N, 10,9 E | e.l. 13.-16.06.[19]34; [Coll. Fiori] | --- |  |  |
| 5770 | MCSNM, Milano | [Italy,] Emilia Romagna, Colli Bolognesi | 44,5 N, 11,2 E | 06.07.[19]43; [Coll. Fiori] | '*italica*' | 2x |  |
| 5771 | MCSNM, Milano | [Italy,] Emilia Romagna, [Passo dei] Mandrioli | 43,8 N, 11,9 E | e.l. 15.01.[19]29; [Coll. Fiori] | '*enigmatica*' |  |  |
| 5772 | MCSNM, Milano | [Italy,] Abruzzo, Tortoreto | 42,8 N, 13,9 E | 15.06.[19]36; [Coll. Fiori] | '*enigmatica*' |  |  |
| 5773 | MCSNM, Milano | [Italy,] Lazio | *41,7 N, 12,9 E* | 02.[19]06; [Coll. Fiori] | '*italica*' |  | *grentzenbergi* |
| 5774 | MRST, Terrasini | [Italy, Sicilia,] Ustica | 38,7 N, 13,2 E | 04.09.1959; Gino Asola [leg.]; [Coll. De Stefani] | --- |  |  |
| 5775 | MRST, Terrasini | [Italy, Sicilia,] Palermo | 38,1 N, 13,3 E | 03.X.1953; [Coll. De Stefani] | --- |  |  |
| 5776 | MRST, Terrasini | [Italy, Sicilia,] Buonfornello | 38,0 N, 13,8 E | 09.06.1956; G. Asola [leg.]; [Coll. De Stefani] | --- |  | *grentzenbergi* |
| 6696 | MRST, Terrasini | [Italy,] Sicilia, Ventimiglia | 37,9 N, 13,6 E | 1940; [Coll. Mariani] | '*melitensis*' ^L^ | 2x |  |
| 6697 | MRST, Terrasini | [Italy,] Sicilia, Casteldaccia | 38,1 N, 13,5 E | Ottobr 1911; [Coll. Mariani] | --- |  |  |
| 6698 | MRST, Terrasini | [Italy, Sicilia,] Palermo | 38,1 N, 13,3 E | asorto [19]51; [Coll. Mariani] | '*italica*' ^L^ |  |  |
| 6699 | MRST, Terrasini | [Italy, Sicilia,] Palermo | 38,1 N, 13,3 E | 20.IX.1951; [Coll.] Mariani | --- |  |  |
| 6700 | MRST, Terrasini | [Italy, Sicilia,] Casteldaccia | 38,1 N, 13,5 E | Maggio 1917; [Coll. Mariani] | --- |  | *grentzenbergi* |
| 6701 | MRST, Terrasini | [Italy, Sicilia,] Palermo | 38,1 N, 13,3 E | 3.VIII.[19]51; [Coll.] Mariani | --- |  |  |
| 6702 | MRST, Terrasini | [Italy,] Sicilia, Casteldaccia | 38,1 N, 13,5 E | Luglio 1911; [Coll. Mariani] | '*italica*' ^HL^ |  |  |
| 7242 | HNHM, Budapest | [Italy,] Calabria, Aspromonte | 38,2 N, 15,9 E | VII 1891; H. Stauder | '*italica*' |  |  |
| 7243 | HNHM, Budapest | [Italy, Campania] Capri | 40,6 N, 14,2 E | [1884] (Staudinger) | --- |  | *grentzenbergi* |
| 7244 | HNHM, Budapest | [Italy, Campania] Capri | 40,6 N, 14,2 E | [1884] (Staudinger) | '*italica*' | 2x | *grentzenbergi* |
| 7257 | ZSM, München | [Italy,] Liguria Occ., San Remo | 43,8 N, 7,8 E | e.l. VIII.[19]25; W. Gieseling [leg.] | *euphorbiae* |  |  |
| 7258 | ZSM, München | [Italy,] Liguria Occ., San Remo | 43,8 N, 7,8 E | e.l. VIII.[19]25; W. Gieseling [leg.] | *euphorbiae* |  |  |
| 7260 | ZSM, München | [Italy,] Liguria, Oz[s]pedaletti | 43,8 N, 7,7 E | 10.VIII.[19]11; [Coll. Daniel, München] | *euphorbiae* |  |  |
| 7264 | ZSM, München | Italia central, [Lazio,] Mti. Simbruini | 41,9 N, 13,2 E | 27.7.[1928]; coll. F. Dannehl, Paratyp "*subiacensis*" | '*italica*' |  | *grentzenbergi* |
| 7265 | ZSM, München | Italia central, [Lazio,] Mti. Simbruini | 41,9 N, 13,2 E | [1928] coll. F. Dannehl, Paratyp "*subiacensis*" | '*enigmatica*' | 2x | *grentzenbergi* |
| 7266 | ZSM, München | Italia central, [Lazio,] Mti. Simbruini | 41,9 N, 13,2 E | [1928] coll. F. Dannehl, Paratyp "*subiacensis*" | --- |  |  |
| 7267 | ZSM, München | [Italy, Lazio] Mont Sabini, (Subiaco) | 42,2 N, 12,8 E | [1928] coll. F. Dannehl, Paratyp "*subiacensis*" | --- |  | *grentzenbergi* |
| 7268 | ZSM, München | It[aly, Lazio,] Rom | 41,9 N, 12,3 E | 1907; [Coll.] Franz Daniel, München | *euphorbiae* ^HL^ |  | *grentzenbergi* |
| 7269 | ZSM, München | It[aly, Campania,] Capri | 40,6 N, 14,2 E | 8.[19]14; Sonn-Bethel [leg.]; ex coll. Dr. Kaiser | '*enigmatica*' |  | *grentzenbergi* |
| 7270 | ZSM, München | It[aly, Campania,] Capri | 40,6 N, 14,2 E | Sept.[19]14; Sonn-Bethel [leg.]; ex coll. Dr. Kaiser | '*enigmatica*' ^HL^ | 2x | *grentzenbergi* |
| 7271 | ZSM, München | [Italy, Campania,] Capri | 40,6 N, 14,2 E | [18]85, 11.6.; Sammlung Daumiller | '*enigmatica*' | 2x | *grentzenbergi* |
| 7272 | ZSM, München | [Italy, Campania,] Capri | 40,6 N, 14,2 E | 14.7.[19]21; Gehlen | --- |  | *grentzenbergi* |
| 7273 | ZSM, München | It[aly, Campania,] Capri | 40,6 N, 14,2 E | e.l. 1.IV.[19]11; Gehlen | *euphorbiae* | 2x | *grentzenbergi* |
| 7274 | ZSM, München | [Italy, Campania,] Capri | 40,6 N, 14,2 E | 1913; Ex Coll. Cl. Hörhammer | '*italica*' |  | *grentzenbergi* |
| 7275 | ZSM, München | [Italy, Campania,] Capri | 40,6 N, 14,2 E | 29.7.[19]25; E.G. Danckwardt, München | *euphorbiae* | 2x | *grentzenbergi* |
| 7276 | ZSM, München | [Italy,] Sicilien, le Madonie | 37,9 N, 14,0 E | 13.-15.V.[19]34; Dr. Eisenberger | '*italica*' | 2x | *grentzenbergi* |
| 7277 | ZSM, München | Mittel-Italien, [Campania,] Sorrent[o] | 40,6 N, 14,4 E | e.l. 10.VIII.[19]33; [Coll.] Daniel, München | '*italica*' |  |  |
| 7278 | ZSM, München | [Italy,] Calabria, Aspromonte | 38,2 N, 15,9 E | VII.1914; H. Stauder [leg.] | '*italica*' |  |  |
| 7279 | ZSM, München | [Italy,] Calabria, Aspromonte | 38,2 N, 15,9 E | VII.1914; H. Stauder [leg.]; [Coll.] Daniel, München | --- |  | *grentzenbergi* |
| 7308 | ZSM, München | [Italy,] Sizilien, Vulcano | 38,4 N, 15,0 E | 31.8.[19]63; Bruno Fischer | '*italica*' |  | *grentzenbergi* |
| 7309 | ZSM, München | [Italy,] Sizilien, Vulcano | 38,4 N, 15,0 E | 1.8.[19]63; Bruno Fischer | '*italica*' |  |  |
| 7310 | ZSM, München | [Italy,] Sizilien, Vulcano | 38,4 N, 15,0 E | 29.7.[19]63; Bruno Fischer | '*italica*' |  |  |
| 7314 | MWM, München | Italien, [Liguria,] San Remo | 43,8 N, 7,8 E | e.l. 7.6.1930; J. Brückl | --- |  | *grentzenbergi* |
| 7315 | MWM, München | Ital[y, Lombardia,] Milano | 45,4 N, 9,2 E | e.l. 26.8.[19]52 | *euphorbiae* |  |  |
| 7316 | MWM, München | Ital[y], Apulien, Golf von Manfredonia | 41,6 N, 15,9 E | 11.7.[19]68, e.l. | *euphorbiae* | 2x |  |
| 7317 | MWM, München | Ital[y], Apulien, Golf von Manfredonia | 41,6 N, 15,9 E | 18.6.[19]68, 22.9. e.l. | *euphorbiae* | 2x |  |
| 7318 | MWM, München | Ital[y], Apulien, Golf von Manfredonia | 41,6 N, 15,9 E | 18.6.[19]68 | '*italica*' |  |  |
| 7319 | MWM, München | Ital[y, Apulia,] Golf von Manfredonia | 41,6 N, 15,9 E | 30.05.1969; leg. Wiegel | '*italica*' |  |  |
| 7320 | MWM, München | Ital[y], Apulien, Golf von Manfredonia | 41,6 N, 15,9 E | e.l. 16.7.1971 | '*italica*' |  | *grentzenbergi* |
| 7321 | MWM, München | Ital[y], Apulien, Golf von Manfredonia | 41,6 N, 15,9 E | 24.6.1971, e.l. | '*italica*' |  | *grentzenbergi* |
| 7322 | MWM, München | [Italy,] Sizilien, Sy[i]racusa | 37,1 N, 15,3 E | 31.8.[19]55; R. Löberbauer | *euphorbiae* | 2x |  |
| 7323 | MWM, München | [Italy,] Sizilien, Sy[i]racusa | 37,1 N, 15,3 E | 31.8.[19]55; R. Löberbauer | '*italica*' |  | *grentzenbergi* |
| 7941 | NHM, London | [Italy,] Sicilia, Umg. Palermo | 38,1 N, 13,3 E | e.l. 19.9.1928; H. Stauder [leg.]; [Coll.] Rothschild | '*melitensis*' | 2x | *grentzenbergi* |
| 7942 | NHM, London | [Italy,] Sicilia, Umg. Palermo | 38,1 N, 13,3 E | e.l. 10.8.1928; H. Stauder [leg.]; [Coll.] Rothschild | '*italica*' | 2x |  |
| 7943 | NHM, London | [Italy,] Sicilia, Umg. Palermo | 38,1 N, 13,3 E | e.l. 5.9.1928; H. Stauder [leg.]; [Coll.] Rothschild | '*italica*' ^BL^ | 2x |  |
| 7944 | NHM, London | [Italy,] Sicilia, Umg. Palermo | 38,1 N, 13,3 E | e.l. 9.9.1928; H. Stauder [leg.]; [Coll.] Rothschild | '*italica*' | 2x | *grentzenbergi* |
| 7945 | NHM, London | [Italy,] Sicilia, Umg. Palermo | 38,1 N, 13,3 E | e.l. 19.8.1928; H. Stauder [leg.]; [Coll.] Rothschild | *euphorbiae* | 2x | *grentzenbergi* |
| 7946 | NHM, London | [Italy,] Sicilia, Umg. Palermo | 38,1 N, 13,3 E | e.l. 21.7.1928; H. Stauder [leg.]; [Coll.] Rothschild | '*italica*' | 2x |  |
| 7950 | NHM, London | [Italy, Campania,] Sorrento, Mte. Faito | 40,7 N, 14,5 E | ex larva VII [19]27; H. Stauder [leg.]; [Coll.] Rothschild | '*italica*' | 2x | *grentzenbergi* |
| 7951 | NHM, London | [Italy, Campania,] Sorrento, Mte. Faito | 40,7 N, 14,5 E | ex larva VII [19]27; H. Stauder [leg.]; [Coll.] Rothschild | '*italica*' | 2x |  |
| 7952 | NHM, London | [Italy, Campania,] Sorrento, Mte. Faito | 40,7 N, 14,5 E | ex larva VII [19]27; H. Stauder [leg.]; [Coll.] Rothschild | '*italica*' | 2x | *grentzenbergi* |
| 7953 | NHM, London | [Italy, Campania,] Sorrento, Mte. Faito | 40,7 N, 14,5 E | ex larva VII [19]27; H. Stauder [leg.]; [Coll.] Rothschild | '*italica*' | 2x | *grentzenbergi* |
| 7954 | NHM, London | [Italy, Sicilia,] Palermo, S. Martino, 500m | 38,1 N, 13,3 E | ex larva VII [19]27; H. Stauder [leg.]; [Coll.] Rothschild | '*italica*' ^L^ |  |  |
| 7955 | NHM, London | [Italy, Sicilia,] Palermo, S. Martino, 500m | 38,1 N, 13,3 E | ex larva VII [19]27; H. Stauder [leg.]; [Coll.] Rothschild | '*italica*' | 2x | *grentzenbergi* |
| 7956 | NHM, London | [Italy, Sicilia,] Palermo, S. Martino, 500m | 38,1 N, 13,3 E | ex larva VII [19]27; H. Stauder [leg.]; [Coll.] Rothschild | '*italica*' ^L^ |  |  |
| 7957 | NHM, London | [Italy, Sicilia,] Palermo, S. Martino, 500m | 38,1 N, 13,3 E | ex larva VII [19]27; H. Stauder [leg.]; [Coll.] Rothschild | '*italica*' | 2x | *grentzenbergi* |
| 7958 | NHM, London | [Italy, Sicilia,] Palermo, S. Martino, 500m | 38,1 N, 13,3 E | ex larva VII [19]27; H. Stauder [leg.]; [Coll.] Rothschild | '*italica*' | 2x |  |
| 7959 | NHM, London | [Italy, Sicilia,] Palermo, S. Martino, 500m | 38,1 N, 13,3 E | ex larva VII [19]27; H. Stauder [leg.]; [Coll.] Rothschild | '*italica*' | 2x |  |
| 7960 | NHM, London | [Italy, Sicilia,] Palermo, S. Martino, 500m | 38,1 N, 13,3 E | ex larva VII [19]27; H. Stauder [leg.]; [Coll.] Rothschild | '*italica*' ^BL^ | 2x | *grentzenbergi* |
| 7961 | NHM, London | [Italy, Sicilia,] Palermo, S. Martino, 500m | 38,1 N, 13,3 E | ex larva VII [19]27; H. Stauder [leg.]; [Coll.] Rothschild | '*italica*' | 2x | *grentzenbergi* |
| 7962 | NHM, London | [Italy, Sicilia,] Palermo, S. Martino, 500m | 38,1 N, 13,3 E | ex larva VII [19]27; H. Stauder [leg.]; [Coll.] Rothschild | '*italica*' | 2x | *grentzenbergi* |
| 7963 | NHM, London | [Italy, Sicilia,] Palermo, S. Martino, 500m | 38,1 N, 13,3 E | ex larva VII [19]27; H. Stauder [leg.]; [Coll.] Rothschild | '*italica*' | 2x |  |
| 7964 | NHM, London | [Italy, Toscana,] Firenze, S. Marcello, Pistoiese | 44,1 N, 10,8 E | 25 Agosto [1]905; Steffanelli [leg.]; [Coll.] Rothschild | '*italica*' | 2x |  |
| 7966 | NHM, London | [Italy,] Liguria, Oz[s]pedale]tti | 43,8 N, 7,7 E | 10/8 1911; Coll. O. Bubacek/Levick | *euphorbiae* |  |  |
| 7967 | NHM, London | Italia, Lig[uria] Occ., Nervia | 44,4 N, 9,0 E | Coll. IX.[19]08; Turati E.; [Coll.] Levick | *euphorbiae* | 2x |  |
| 7970 | NHM, London | [Italy,] Calabria, Aspromonte | 38,2 N, 15,9 E | VII.1914; H. Stauder [leg.]; [Coll.] Rothschild | '*italica*' | 2x |  |
| 7971 | NHM, London | [Italy,] Calabria, Aspromonte (800m) | 38,2 N, 15,9 E | Ex larva 20.9.[19]20; H. Stauder [leg.]; [Coll.] Rothschild | '*italica*' |  |  |
| 7989 | NHM, London | [Italy,] Sicilia, Porto Palo | 37,6 N, 12,9 E | 16.06.1927; [Coll.] Rothschild | '*italica*' | 2x |  |
| 7990 | NHM, London | [Italy, Campania,] Capri | 40,6 N, 14,2 E | 1904; R. South Coll. | --- |  |  |
| 7999 | NHM, London | [Italy,] Sicilia, Porto Palo | 37,6 N, 12,9 E | 16.06.1927; [Coll.] Rothschild | *euphorbiae* | 2x |  |
| 8004 | NHM, London | Malta | 35,9 N, 14,4 E | ex Larva 1980; A.R. Pittaway [leg.]; Cadiou Coll. | *tithymali* | 2x |  |
| 8006 | NHM, London | [Italy, Sicilia,] Liparische Inseln, Vulcano | 38,4 N, 15,0 E | 18.11.[19]76., e.o.; E. Loser [leg.]; Cadiou Coll. | '*italica*' | 2x | *grentzenbergi* |
| 8008 | NHM, London | Italien, [Sicilia,] Insel Vulcano | 38,4 N, 15,0 E | e.o. 18.11.1974; Rüttimann [leg.]; Cadiou Coll. | '*italica*' | 2x |  |
| 8009 | NHM, London | Italien, [Sicilia,] Insel Vulcano | 38,4 N, 15,0 E | e.o. 20.9.1974; Rüttimann [leg.]; Cadiou Coll. | '*italica*' | 2x |  |
| 8010 | NHM, London | [Italy,] Sizilien, Catania | 37,5 N, 15,0 E | Mai 1964; leg. Leinfest; Cadiou Coll. | '*italica*' | 2x |  |
| 8011 | NHM, London | [Italy,] Sizilien, Catania | 37,5 N, 15,0 E | Mai 1964; leg. Leinfest; Cadiou Coll. | '*italica*' | 2x |  |
| 8018 | NHM, London | [Italy, Campania,] Capri | 40,6 N, 14,2 E | 1894; Cadiou Coll. | --- |  | *grentzenbergi* |
| 8019 | NHM, London | [Italy, Campania,] Capri | 40,6 N, 14,2 E | 1899; Cadiou Coll. | *euphorbiae* ^L^ | 2x |  |
| 8639 | NMNH, Malta | Malta | 35,9 N, 14,4 E | 14.7.[19]54 e.l.; Coll. Lanfranco | --- |  |  |
| 8640 | NMNH, Malta | Malta | 35,9 N, 14,4 E | 26.4.195[?]; Coll. Lanfranco | --- |  |  |
| 8641 | NMNH, Malta | Malta | 35,9 N, 14,4 E | 11.9.[19]54; Coll. Lanfranco | --- |  |  |
| 8642 | NMNH, Malta | Malta | 35,9 N, 14,4 E | 9.[??].[19]54, e.l.; Coll. Lanfranco | --- |  |  |
| 8643 | NMNH, Malta | Malta, Wied il-Kbir | 35,9 N, 14,5 E | 3.11.[19]64, e.l.; Coll. Lanfranco | --- |  |  |
| 8644 | NMNH, Malta | Malta | 35,9 N, 14,4 E | 26.6.[19]54, e.l.; Coll. Lanfranco | --- |  |  |
| 8645 | NMNH, Malta | Malta | 35,9 N, 14,4 E | 2.9.[19]54, e.l.; Coll. Lanfranco | --- |  |  |
| 8646 | NMNH, Malta | Malta | 35,9 N, 14,4 E | 4.9.[19]54, e.l.; Coll. Lanfranco | --- |  |  |
| 8647 | NMNH, Malta | Malta | 35,9 N, 14,4 E | 10.10.[19]53, e.l.; Coll. Lanfranco | --- |  |  |
| 8648 | NMNH, Malta | Malta, Wied il-Kbir | 35,9 N, 14,5 E | 1.11.[19]64, e.l.; Coll. Lanfranco | --- |  |  |
| 8649 | NMNH, Malta | Malta | 35,9 N, 14,4 E | 6.9.[19]54, e.l.; Coll. Lanfranco | *tithymali* ^L^ | 2x |  |
| 8650 | NMNH, Malta | Malta | 35,9 N, 14,4 E | 7.8.[19]54, e.l.; Coll. Lanfranco | --- |  |  |
| 8651 | NMNH, Malta | Malta | 35,9 N, 14,4 E | XI.1964, e.l.; Coll. Dr. Carmelo Delucca | *euphorbiae* | 2x |  |
| 8652 | NMNH, Malta | Malta, Gharghur | 35,9 N, 14,5 E | 30.IX.1968 collected; Coll. Dr. Carmelo Delucca | --- |  |  |
| 8653 | NMNH, Malta | Malta, Gharghur | 35,9 N, 14,5 E | 25.IX.1968 collected; Coll. Dr. Carmelo Delucca | --- |  |  |
| 8654 | NMNH, Malta | Malta, Paolo | 35,9 N, 14,5 E | 18.IX.1981; M. Zammit leg. et Coll. | --- |  |  |
| 8655 | pcAC, Malta | Malta, Gzira | 35,9 N, 14,5 E | 28.XI.1986; Aldo Catania leg. | --- |  |  |
| 8656 | pcAC, Malta | Malta, Gzira | 35,9 N, 14,5 E | 14.IX.1983; Aldo Catania leg. | --- |  |  |
| 8657 | pcPS, Malta | Malta, Rabat | 35,9 N, 14,4 E | IX.1965; Paul Sammut leg. | --- |  |  |
| 8658 | pcPS, Malta | Malta | 35,9 N, 14,4 E | X.1982, e.l.; Paul Sammut leg. | --- |  |  |
| 8659 | pcPS, Malta | Malta, Rabat | 35,9 N, 14,4 E | 14.VI.1983; Paul Sammut leg. | --- |  |  |
| 8660 | SMNS, Stuttgart | Italien, [Abruzzo,] Villa Rosa (Adria) | 42,9 N, 13,9 E | Aug. [19]67; Sammlung Langer | '*italica*' |  |  |
| 8661 | SMNS, Stuttgart | Italien, [Abruzzo,] Villa Rosa (Adria) | 42,9 N, 13,9 E | Aug. [19]67; Sammlung Langer | '*italica*' |  |  |
| 8662 | SMNS, Stuttgart | Italien, [Puglia,] Gargano, Peschici | 41,9 N, 16,0 E | 30.8.[19]70; Sammlung Langer | '*italica*' |  |  |
| 8663 | SMNS, Stuttgart | Italien, [Puglia,] Gargano, Manacore | 41,9 N, 16,0 E | 4.7.[19]76; Sammlung Langer | '*italica*' |  |  |
| 8664 | SMNS, Stuttgart | [Italy,] Sizilien, Palermo, St. Martino della Scale | 38,1 N, 13,3 E | 12.5.-20.5.[19]64; leg. Hauck; Coll. A. Langheinrich | '*italica*' |  |  |
| 8665 | SMNS, Stuttgart | [Italy,] Sizilien, Palermo, St. Martino della Scale | 38,1 N, 13,3 E | 12.5.-20.5.[19]64; leg. Hauck; Coll. A. Langheinrich | '*italica*' |  |  |
| 8666 | SMNS, Stuttgart | [Italy,] Sizilien, Palermo, St. Martino della Scale | 38,1 N, 13,3 E | 12.5.-20.5.[19]64; leg. Hauck; Coll. A. Langheinrich | '*italica*' |  |  |
| 8667 | SMNS, Stuttgart | [Italy,] Sizilien, Palermo, St. Martino della Scale | 38,1 N, 13,3 E | 12.5.-20.5.[19]64; leg. Hauck; Coll. A. Langheinrich | '*italica*' |  | *grentzenbergi* |
| 8668 | SMNS, Stuttgart | [Italy,] Sizilien, Palermo, St. Martino della Scale | 38,1 N, 13,3 E | 12.5.-20.5.[19]64; leg. Hauck; Coll. A. Langheinrich | '*italica*' |  |  |
| 8669 | SMNS, Stuttgart | [Italy, Sicilia,] Palermo | 38,1 N, 13,3 E | Aug. 1965; Hauck [leg.]; Coll. Dr. Weinmann | '*italica*' |  | *grentzenbergi* |
| 8670 | ZFMK, Bonn | [Italy, Campania,] Capri | 40,6 N, 14,2 E | 8.[19]14; Bonn-Rethel | '*enigmatica*' ^L^ |  | *grentzenbergi* |
| 8671 | ZFMK, Bonn | [Italy, Campania,] Capri | 40,6 N, 14,2 E | e.l. IV.1909 | *euphorbiae* ^HL^ |  | *grentzenbergi* |
| 8672 | ZFMK, Bonn | [Italy, Campania,] Capri | 40,6 N, 14,2 E | 1909 | --- |  | *grentzenbergi* |
| 8673 | ZFMK, Bonn | [Italy,] Sicilia, Umg. Palermo | 38,1 N, 13,3 E | 1/9.1928 e.l.; H. Stauder [leg.] | *euphorbiae* |  |  |
| 8674 | ZFMK, Bonn | [Italy,] Sicilia, Umg. Palermo | 38,1 N, 13,3 E | 7.8.1928, e.l.; H. Stauder [leg.] | '*italica*' |  | *grentzenbergi* |
| 8675 | ZFMK, Bonn | [Italy,] Sicilia, Umg. Palermo | 38,1 N, 13,3 E | 4.8.1928, e.l.; H. Stauder [leg.] | *euphorbiae* |  |  |
| 8676 | ZFMK, Bonn | Süd-Italien | *41,2 N, 15,2 E* | Juni 1920; Boppard | *euphorbiae* |  | *grentzenbergi* |
| 8677 | ZFMK, Bonn | Süd-Italien | *41,2 N, 15,2 E* | Juni 1920; Boppard | '*enigmatica*' |  | *grentzenbergi* |
| 8678 | ZFMK, Bonn | [Italy, Campania,] Capri | 40,6 N, 14,2 E | Sept. [19]14; Bonn-Rethel | --- |  | *grentzenbergi* |
| 8895 | ZFMK, Bonn | [Italy, Lazio,] Prov. di Roma, Subiaco | 41,9 N, 13,1 E | August 1909 | *tithymali* |  | *grentzenbergi* |
