# Supplementary material for: Mitochondrial lineage sorting in action – historical biogeography of the Hyles euphorbiae complex (Sphingidae, Lepidoptera) in Italy
Source: BMC Evol Biol. 2013 Apr 18;13:83. doi: 10.1186/1471-2148-13-83 (PMC3655913; doi:10.1186/1471-2148-13-83)
Supplement: Additional file 2: Table S2 — Variable sites of the three amplicons used in this study for the Mediterranean lineages of the HEC. Consensus sequences for each Mediterranean HEC lineage of all individuals from Hundsdoerfer et al. [31] were reduced to variable sites of the three fragments B, H, L. Character states for the positive control outgroup H. livornica are reported for comparison. Positions are numbered according to the 2284 bp alignment of COI/II genes of Hundsdoerfer et al. [31]; fragment B: position 89–365, fragment H: 1094–1373, fragment L: 1909–2145. Round brackets indicate that not all individuals of the lineage bear the substitution; lowercases indicate rare substitutions in only few or single specimens. [file 1471-2148-13-83-S2.doc]

| **position:** | **120** | **123** | **129** | **150** | **153** | **156** | **158** | **164** | **171** | **180** | **189** | **192** | **235** | **264** | **273** | **281** | **288** | **309** | **316** | **321** |  |  |  |  |  |  |  |
| --- | --- | --- | --- | --- | --- | --- | --- | --- | --- | --- | --- | --- | --- | --- | --- | --- | --- | --- | --- | --- | --- | --- | --- | --- | --- | --- | --- |
|  | C | A | T | T | T | T | C | T | A | T | T | C | T | T | T | T | T | C | T | T |  |  |  |  |  |  |  |
| ***euphorbiae*** | . | C | . | .(c) | . | C | . | (g) | . | . | . | (T) | . | (c) | . | . | . | . | . | . |  |  |  |  |  |  |  |
| ***tithymali*** | . | . | (c) | . | . | . | . | (g) | . | . | (c) | . | (c) | . | C | (a) | (a) | (t) | . | (c) |  |  |  |  |  |  |  |
| **'*melitensis*'** | . | C | . | . | . | . | T | . | . | C | C | . | . | . | . | . | . | . | C | . |  |  |  |  |  |  |  |
| **'*italica*'** | . | . | . | . | . | . | . | . | . | . | . | . | . | . | . | (a) | . | . | . | . |  |  |  |  |  |  |  |
| ***cretica*** | . | . | . | . | C | . | . | . | . | . | . | . | . | . | . | . | . | . | . | . |  |  |  |  |  |  |  |
| **'*enigmatica*'** | . | . | . | . | . | . | . | . | (g) | . | . | . | . | . | C | . | . | . | . | . |  |  |  |  |  |  |  |
| ***H. livornica* (+)** | T | T | . | . | . | . | . | . | . | . | . | . | . | . | . | . | . | . | . | . |  |  |  |  |  |  |  |
|  |  |  |  |  |  |  |  |  |  |  |  |  |  |  |  |  |  |  |  |  |  |  |  |  |  |  |  |
| **position:** | **1116** | **1119** | **1122** | **1125** | **1131** | **1143** | **1164** | **1165** | **1198** | **1202** | **1203** | **1209** | **1212** | **1219** | **1230** | **1245** | **1251** | **1263** | **1269** | **1278** | **1287** | **1291** | **1296** | **1311** | **1320** | **1332** | **1350** |
|  | T | C | T | C | T | A | A | G | C | C | T | T | A | T | A | T | A | T | T | T | T | G | A | T | C | T | T |
| ***euphorbiae*** | . | . | . | (t) | . | . | (G) | (a) | (t) | (t) | . | (c) | . | (c) | (G) | . | (g) | . | . | (C) | . | (a) | (t) | (C) | (t) | . | . |
| ***tithymali*** | (c) | . | . | . | . | . | . | . | . | . | . | . | . | . | (G) | . | . | . | . |  | . | . | . | C | (T) | . | . |
| **'*melitensis*'** | C | . | . | T | . | . | . | . | T | G | . | . | . | . | . | . | . | . | . | C | . | . | . | . | . | . | . |
| **'*italica*'** | C | (T) | . | . | . | . | . | . | . | . | . | . | . | . | (G) | (C) | . | (c) | . | . | . | . | . | C | (t) | . | . |
| ***cretica*** | . | . | . | . | . | . | (G) | . | . | . | . | . | . | . | G | . | . | . | . | . | . | . | . | . | . | . | . |
| **'*enigmatica*'** | . | . | . | . | . | (G) | . | . | . | . | . | . | . | . | . | . | . | . | . | . | . | . | . | . | (T) | C | . |
| ***H. livornica* (+)** | C | T | C | T | C | . | . | . | T | . | A | . | T | . | . | . | . | . | C | C | A | . | . | . | T | . | C |
|  |  |  |  |  |  |  |  |  |  |  |  |  |  |  |  |  |  |  |  |  |  |  |  |  |  |  |  |
| **position:** | **1938** | **1961** | **1967** | **1970** | **1979** | **1985** | **1986** | **1988** | **1997** | **2015** | **2039** | **2042** | **2081** | **2084** | **2090** | **2093** | **2108** | **2123** |  |  |  |  |  |  |  |  |  |
|  | C | T | T | T | A | C | C | C | C | T | C | C | C | A | T | C | A | T |  |  |  |  |  |  |  |  |  |
| ***euphorbiae*** | . | . | . | . | . | . | (t) | (a/t) | (t) | (c) | T | (t) | . | . | . | (T) | . | . |  |  |  |  |  |  |  |  |  |
| ***tithymali*** | . | . | . | . | (G) | (t) | . | . | . | . | . | (t) | . | . | . | . | (g) | . |  |  |  |  |  |  |  |  |  |
| **'*melitensis*'** | T | . | C | . | . | T | . | . | . | . | T | T | . | . | . | T | . | . |  |  |  |  |  |  |  |  |  |
| **'*italica*'** | . | . | . | . | (G) | . | . | T | . | . | . | . | . | T | (c) | . | . | (c) |  |  |  |  |  |  |  |  |  |
| ***cretica*** | . | . | . | . | G | . | . | T | . | . | . | . | . | . | . | . | . | . |  |  |  |  |  |  |  |  |  |
| **'*enigmatica*'** | . | C | . | . | . | . | . | . | . | . | . | T | . | . | . | . | . | . |  |  |  |  |  |  |  |  |  |
| ***H. livornica* (+)** | . | . | . | A | . | T | T | T | T | . | T | . | T | . | . | T | . | . |  |  |  |  |  |  |  |  |  |
